# Functional Interplay between Methyltransferases and Inflammasomes in Inflammatory Responses and Diseases

**DOI:** 10.3390/ijms22147580

**Published:** 2021-07-15

**Authors:** Young-Su Yi

**Affiliations:** Department of Life Sciences, Kyonggi University, Suwon 16227, Korea; ysyi@kgu.ac.kr; Tel.: +82-31-249-9644

**Keywords:** methyltransferase, methylation, epigenetics, inflammasome, DNA, histone

## Abstract

An inflammasome is an intracellular protein complex that is activated in response to a pathogenic infection and cellular damage. It triggers inflammatory responses by promoting inflammatory cell death (called pyroptosis) and the secretion of pro-inflammatory cytokines, interleukin (IL)-1β and IL-18. Many types of inflammasomes have been identified and demonstrated to play a central role in inducing inflammatory responses, leading to the onset and progression of numerous inflammatory diseases. Methylation is a biological process by which methyl groups are transferred from methyl donors to proteins, nucleic acids, and other cellular molecules. Methylation plays critical roles in various biological functions by modulating gene expression, protein activity, protein localization, and molecular stability, and aberrant regulation of methylation causes deleterious outcomes in various human diseases. Methylation is a key determinant of inflammatory responses and diseases. This review highlights the current understanding of the functional relationship between inflammasome regulation and methylation of cellular molecules in inflammatory responses and diseases.

## 1. Introduction

Inflammation is an innate immune response that protects the body from microbial infection and cellular stress [1,2]. The inflammatory response relies on pattern-recognition receptors (PRRs) by recognition of a variety of pathogen-associated molecular patterns (PAMPs) and danger-associated molecular patterns (DAMPs) through receptor-ligand interaction [1,2]. The inflammatory response comprises two steps: priming and triggering. Priming is a preparatory step in the inflammatory response, and involves the inducing of the transcription of pro-inflammatory molecules. It occurs via the cell surface and endosomal PRRs, such as toll-like receptors (TLRs) that activate nuclear factor-kappa B (NF-κB), activator protein-1 (AP-1), and interferon (IFN)-regulatory factors (IRFs) [3,4,5]. Triggering, on the other hand, is an activation step of the inflammatory response, which involves the activation of inflammasomes, intracellular protein complexes comprising PRRs and inflammatory molecules and typically occurs via intracellular PRRs, such as the nucleotide-binding oligomerization domain (NOD)-like receptors (NLRs), absent in melanoma 2 (AIM2), and inflammatory caspases, mouse caspase-11, human caspase-4, and caspase-5 [6,7,8,9,10,11]. Inflammasome activation is a key feature of the triggering step of inflammatory responses, and dysregulation of inflammasome activation has been considered the key risk factor for chronic inflammation and numerous human diseases [12,13,14,15,16].

Posttranslational modifications (PTMs) are critical determinants of innate immunity and inflammatory responses [17,18]. The functional cooperation between inflammasomes and PTMs has been demonstrated to play pivotal roles in various biological functions. PTMs occur in the components of inflammasomes and regulate the activities and functions of inflammasomes [19,20,21].

Methylation is a biological process by which methyl groups are added to a subset of proteins and nucleic acids, which leads to the epigenetic modifications of these cellular molecules. These molecules achieve the functions required for cellular homeostasis via methylation; therefore, changes in the methylation status have been implicated in various biological conditions and human diseases, including inflammatory responses and diseases. Previous studies have demonstrated the roles of methyltransferases (MTs) in this context, as well as the potential therapeutic strategies that could target these enzymes [22,23,24,25,26]. Different criteria, including substrates and structural features, have been used to classify MTs. Protein MTs (PMTs) methylate proteins; although histone proteins have been identified as the predominant substrates of PMTs at lysine and arginine residues [27,28,29,30,31,32], emerging studies suggest that non-histone proteins are also substrates of PMTs [33,34,35,36]. DNA MTs (DNMTs) methylate DNA by catalyzing CpG methylation in the promoter regions of many genes responsible for various biological functions and human diseases. Accordingly, dysregulated DNA methylation has been considered a major risk factor for aberrant gene expression and human diseases [23,37,38,39,40,41]. MTs also catalyze the methylation of other biological and chemical molecules, such as natural products, organic compounds, chemical elements, and phospholipids, resulting in the modulation of their biological functions [42,43,44,45,46]. Given the roles of MTs, MTs are thought to play pivotal roles in the cooperation of inflammasomes in inflammatory responses and diseases.

This review discusses the functional relationship and interplay between inflammasomes and MTs and assists in better understanding the cooperative roles of these molecules in inflammatory responses and diseases. This review will also provide insight into the development of anti-inflammatory therapeutics that selectively target inflammasomes and MTs.

## 2. Methyltransferases

**Figure 1:**
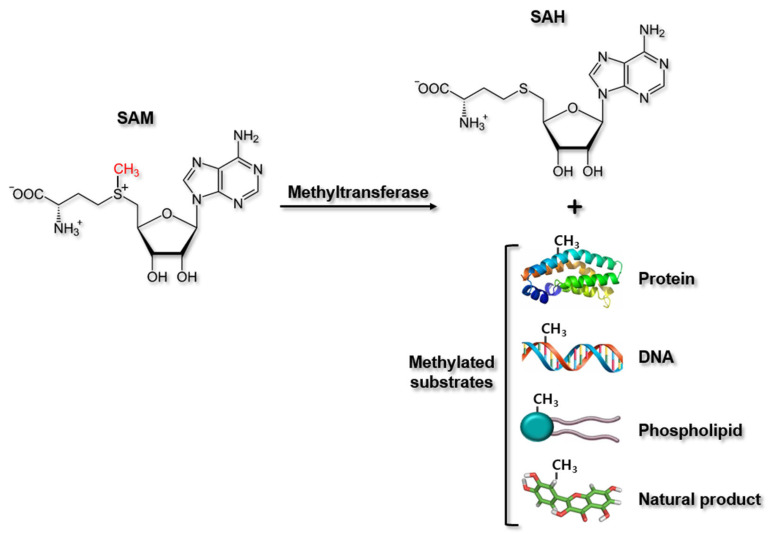
Methylation reaction by methyltransferases. Methyltransferases catalyzes the methylation of cellular molecules, such as proteins, DNA, and phospholipids and some natural products by transferring methyl groups from methyl donor, SAM to their specific substrates, producing methylated substrates and SAH. SAM, *S*−*adenosyl*−*_L_*−*methionine*; SAH, *S−adenosyl*−*_L_*−*homocysteine*.

MTs comprise a large group of enzymes that transfer methyl groups from methyl donors to their substrates. These enzymes share a general mechanism by which MT binds with *S-adenosyl-l-methionine* (SAM) (the universal methyl donor for MTs) as well as the target substrate by the different mechanisms, and transfers a methyl group from SAM directly to the target substrate via the classic SN2 reaction. During this process, SAM is converted to *S-adenosyl-l-homocysteine* (SAH), and the methylated substrates are generated (Figure 1) [47]. MTs are classified using different criteria. Most common way to classify MTs is based on their substrates, such as PMTs, DNMTs, phospholipid MTs, and natural product MTs (Figure 1). MTs are also classified based on their structural features. Here, class I MTs bind SAM using a Rossman fold motif, whereas class II MTs contain a SET (Enhancer-of-zeste and Trithorax) domain for binding SAM. Class III methyltransferases are membrane-associated [48].

### 2.1. DNA Methyltransferases

DNA methylation is a major regulatory process of chromatin structural modification that plays a pivotal role in modulating gene expression during a variety of biological activities. DNA methylation is an essential epigenetic mechanism that functions in combination with other epigenetic changes, such as histone methylation [49,50]. DNA methylation occurs only at cytosine residues in eukaryotic cells, and at both cytosine and adenosine residues in bacteria [51]. Although DNA methylation is generally observed at the palindromic CpG sites of both sense and anti-sense DNA strands, reports have described single-stranded DNA methylation at non-CpG sites [51,52,53]. Correlations of changes in the epigenetic status of DNA with several human diseases, including immune-related diseases, cancers, neurodegenerative diseases, diabetes, and infectious diseases, have been suggested [54,55,56,57,58].

**Figure 2:**
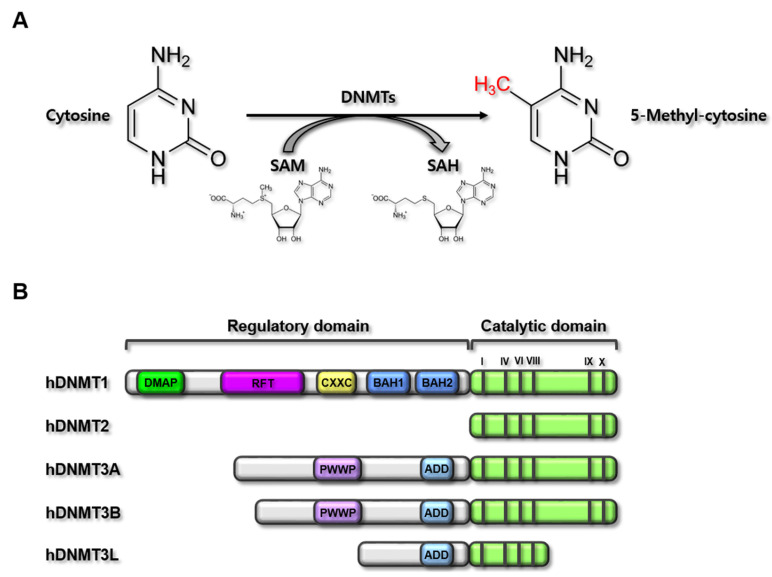
DNMT-mediated methylation reaction and domain structure of human DNMTs. (**A**) Chemical reaction of DNMT-mediated methylation. DNMTs catalyze the methylation at cytosines of CpG in the gene promoter regions by transferring methyl groups from methyl donor, SAM, producing 5-methyl-cytosine in the gene promoter regions and SAH. (**B**) Domain structures of human DNMT (hDNMT) family members. hDNMTs consist of five members; hDNMT1 (1616 amino acids), hDNMT2 (391 amino acids), hDNMT3A (912 amino acids), hDNMT3B (853 amino acids), and hDNMT3L (386 amino acids). hDNMTs have two main domains; the regulatory and catalytic domains. The catalytic domain is conserved in all hDNMTs. hDNMT1 is the longest member with DMAP, RFTD, CXXC, and two BAHs in the regulatory domain. hDNMT2 is the smallest member existing only catalytic domain. hDNMT3A and hDNMT3B have PWWP and ADD in the regulatory domain in common, but hDNMT3B is little bit smaller than hDNMT3A. hDNMT3L has only ADD in the regulatory domain with the shorter catalytic domain. Bars in the catalytic domain (I, IV, VI, VIII, IX, and X) represent the catalytic active sites. SAM, *S-adenosyl-l-methionine*; SAH, *S-adenosyl-l-homocysteine*; DNMT, DNA methyltransferase; DMAP, DNA methyltransferase-associated protein 1-interacting domain; RFT, replication foci targeting sequence domain; CXXC, CXXC domain; BAH, bromo-adjacent homology domain; PWWP, PWWP domain; ADD, ATRX-DNMT3-DNMT3L domain.

DNMTs catalyze DNA methylation by depositing a methyl group on the C-5 position of a cytosine (Figure 2A). Five human DNMTs with highly conserved catalytic motifs were identified: DNMT1, DNMT2, DNMT3A, DNMT3B, and DNMT3L (Figure 2B). The canonical DNMTs (DNMT1, DNMT3A, and DNMT3B) catalyze DNA methylation, whereas DNMT2 and DNMT3L are non-canonical DNMTs that are not catalytically able to methylate DNA [23]. Conserved DNMTs have also been identified in plants with more extensive DNMT gene sequence variations [59], and DNMT4, DNMT5, and DNMT6 have been discovered in fungi and algae [60,61]. During DNA replication, DNA methylation can be reversed by active or passive demethylation processes; however, DNMT1, which is also known as a maintenance DNMT, induces hemimethylation at the CpG sites of the DNA strand that lose methylation during DNA replication. In contrast, DNMT3A and DNMT3B do not exert hemimethylation activity at CpG sites, but instead generate de novo DNA methylation patterns during development and early germ cell differentiation [37].

DNMTs are major epigenetic modulators of the genes expressed in various physiological functions, suggesting that DNMTs could be involved in the pathogenesis of human diseases. Several studies have reported the regulatory roles of DNMTs in human diseases. Interestingly, mutations in DNMT genes have been reported in human diseases. DNMT1 mutation has been identified in neurological diseases [62,63], and DNMT3A mutation was observed in Tatton–Brown–Rahman syndrome, which involves overgrowth and intellectual disability [64]. DNMT3B mutation has been described in several diseases, including immunodeficiency, facial abnormality syndrome, and centromeric instability [65,66]. Many reports have described the roles of DNMTs in cancers, as epigenetic changes in DNA methylation patterns comprise one of the most significant molecular alterations associated with tumorigenesis [67,68]. Therefore, DNMT mutation repair may be a potential approach to the treatment of various human diseases. The modulation of DNMTs via approved DNMT inhibitors, such as 5-azacytidine and 2ʹ-deoxy-5-azacytidine [69,70], could also be a promising cancer epigenome-targeting strategy for cancer treatment. Further investigations of the functional roles of specific DNMT activities in other human disorders, including inflammatory, metabolic, cardiovascular, and neurological diseases, are urgently needed.

### 2.2. Protein Methyltransferases

**Figure 3:**
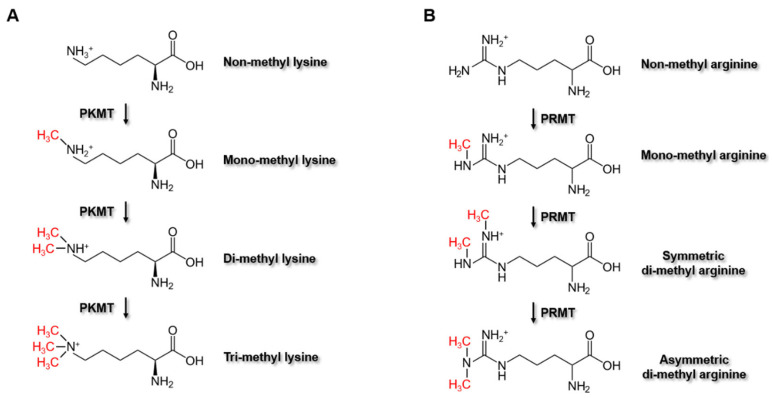
Methylation reactions by PKMT and PRMT. (**A**) Chemical reactions of PKMT-mediated methylation. PKMTs catalyze the methylation at lysine residue of proteins, producing mono-methyl lysine, di-methyl lysine, and tri-methyl lysine. (**B**) Chemical reactions of PRMT-mediated methylation. PRMTs catalyze the methylation at arginine residue of proteins, producing mono-methyl arginine and either of symmetric di-methyl arginine or asymmetric di-methyl arginine. PKMT, protein-lysine methyltransferases; PRMT, protein-arginine methyltransferases.

PMTs catalyze the methylation of target proteins at the nitrogen-containing side chains of lysine, arginine, and histidine [71,72], but also at the carboxyl group of the prenylated cysteine [73,74] by transferring methyl groups from methyl donors. Although these several amino acids have been identified as methylation residues of target proteins by PMTs, most of the studies have been focusing on the PMTs that methylate lysine and arginine residues. This family of protein-lysine MTs (PKMTs) and protein-arginine MTs (PRMTs) comprises more than 60 members, which mediate the methylation of histone or non-histone substrates on lysine or arginine residues, respectively. PKMTs can transfer up to three methyl groups to the side-chain nitrogen of lysine, thus generating four different states of lysine: non-methyl, mono-methyl, di-methyl, or tri-methyl lysine (Figure 3A). Similarly, PRMTs transfer methyl groups to one or both of the two side-chain nitrogens of arginine, thus producing four different states of arginine: non-methyl, mono-methyl, symmetric di-methyl, or asymmetric di-methyl arginine (Figure 3B).

Histones have received a great deal of attention among the known target proteins of MTs [71,75]; as these proteins are essential components of nucleosomes, the regulation of histone methylation plays pivotal roles in gene expression in various biological processes. Moreover, the enzymatic actions of PMTs are thought to be crucial modulators of gene expression, and many studies have demonstrated the active involvement of these PMTs in the pathogenesis of various human diseases, including inflammatory and neurodegenerative diseases, cancers, and other conditions [71,76,77,78,79,80,81,82]. Therefore, PKMTs and PRMTs have elicited substantial interest in the fields of medicinal chemistry and drug discovery through attempts to prevent and treat these diseases, and several anticancer drugs that target MTs have already been developed [71].

## 3. Inflammasomes

Inflammasomes are intracellular protein complexes that induce inflammatory responses and consist of the PRRs localized in the cells and the inflammatory effector molecules, such as inflammatory caspases [7,83,84]. Intracellular PRRs recognize specific PAMPs and DAMPs and, subsequently, form inflammasomes by interacting with inflammatory effector molecules with or without the help of the bipartite adaptor, apoptosis-associated speck-like protein containing a caspase recruitment domain (ASC), leading to inflammasome activation and inflammasome-induced inflammatory responses [7,83,84]. The classification of inflammasomes and the signaling pathways of inflammasome-activated inflammatory responses will be discussed in future studies.

### 3.1. Classification and Molecular Architecture of Inflammasomes

Several different types of inflammasomes can be named after PRRs that promote their assembly. PRRs are distinguished by their structure and assembled into inflammasomes by recognizing different types of PRR-specific PAMPs and DAMPs [7,85]. Inflammasomes are classified into two main groups: canonical and non-canonical inflammasomes [7,85]. Canonical inflammasomes include the NLR family, pyrin, and AIM2 inflammasomes, whereas non-canonical inflammasomes include caspase-11, caspase-4, and caspase-5 inflammasomes [7,85].

**Figure 4:**
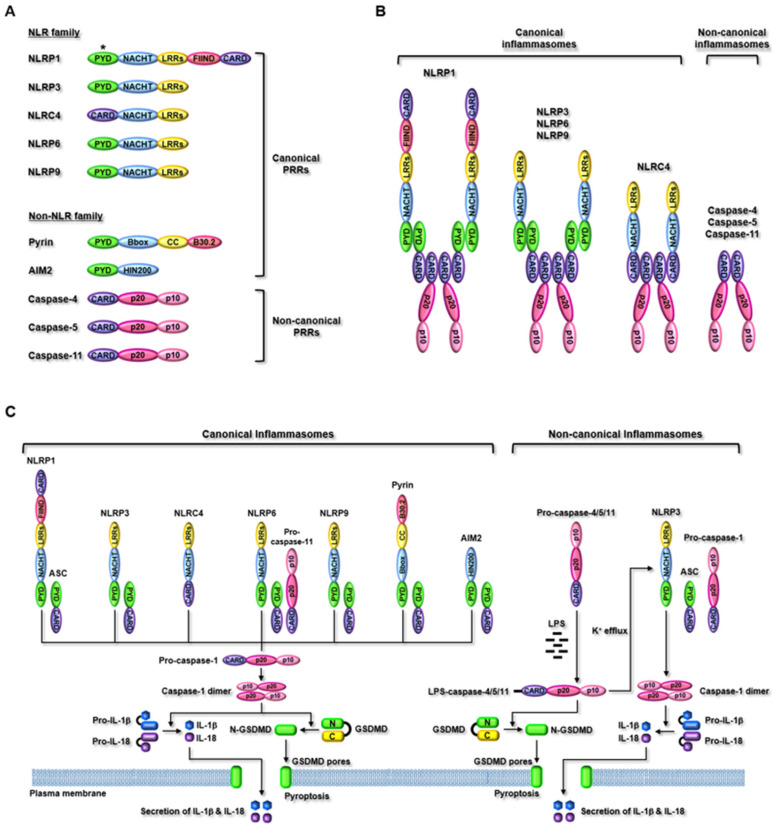
Inflammasome-activated inflammatory signaling (**A**) the domain structure of PRRs. Inflammasome PRRs are intracellular sensors that include PYD and/or CARD. The PRRs also include NACHT, LRRs, and FIIND in NLR family members. Pyrin includes Bbox, CC, and B30.2 and AIM2 includes HIN200. Caspase-4/5/11 have the same domain structure that includes CARD, p20, and p10. *Absence in mouse NLRP1 isoforms (**B**) The structure of inflammasomes. Inflammasome PRRs that include a PYD, such as human NLRP1, NLRP3, NLRP6, NLRP9, pyrin, and AIM2 recruit a bipartite adaptor, ASC to mediate CARD-CARD interactions with pro-caspase-1 (ASC-positive scaffolds). While inflammasome PRRs that include CARD instead of PYD, such as mouse NLRP1b and NLRC4 interact directly with pro-caspase-1 without the help of ASC (ASC-negative scaffolds). PRRs of non-canonical inflammasomes, such as mouse caspase-11, human caspase-4, and caspase-5 that include CARD sense directly intracellular LPS and mediate CARD–CARD interaction without the binding of ASC and pro-caspase-1. (**C**) Inflammasome-activated inflammatory signaling pathways. Inflammasomes are activated in response to PAMPs and DAMPs. The PRRs of canonical inflammasomes directly interact with their specific ligands and respond to various cellular danger signals. The canonical inflammasomes activate caspase-1 by the proteolytic cleavage of CARD and produce active caspase-1 dimers. Activated caspase-1 induces proteolytic cleavage of GSDMD to generate N-GSDMD that then generates GSDMD pores and induces pyroptosis. Activated caspase-1 also induces proteolytic maturation of IL-1β and IL-18 into their active forms, which are secreted through GSDMD pores. PRRs of non-canonical inflammasomes (mouse caspase-11, human caspase-4, and caspase-5) directly interact with intracellular LPS derived from Gram-negative bacteria. The activation of non-canonical inflammasomes also induces proteolytic cleavage of GSDMD, leading to pyroptosis. Non-canonical inflammasomes activate NLRP3 canonical inflammasome by facilitating K^+^ efflux, which subsequently induces proteolytic maturation and secretion of IL-1β and IL-18 through GSDMD pores. PYD, pyrin domain; CARD, caspase recruitment domain; NACHT, nucleotide-binding and oligomerization domain, LRRs, leucine-rich repeats, FIIND, a functional-to-find domain; Bbox, B-box-type zinc finger; CC, coiled-coil; AIM2, absent in melanoma 2; HIN200, hematopoietic interferon-inducible nuclear protein 200; ASC, apoptosis-associated speck-like protein containing a caspase recruitment domain; GSDMD, gasdermin D; LPS, lipopolysaccharide.

NLR family PRRs are subclassified into five different subfamilies: NLRA, NLRB, NLRC, NLRP, and NLRX [86,87,88]. To date, 14 NLRP (NLRP1–14) and five NLRC subfamily members (NLRC1–4) have been identified [86,87,88], but only some subfamily members, such as NLRP1, NLRP3, NLRC4, NLRP6, and NLRP9 have been demonstrated to form inflammasomes and activate inflammasome-induced inflammatory responses [7]. NLRP1 is the first NLR family PRR identified and demonstrated to assemble inflammasomes [89]. NLRP1 has the most complex structure comprising an N-terminal PYD, a nucleotide-binding and oligomerization domain (NACHT), leucine-rich repeats (LRRs), a functional-to-find domain (FIIND), and a C-terminal caspase recruitment domain (CARD) (Figure 4A). Unlike human NLRP1, three isoforms of NLRP1 (NLRP1a, NLRP1b, and NLRP1c) have been discovered in mice; however, an N-terminal PYD in human NLRP1 is absent in mouse NLRP1 (Figure 4A). This provides a different mechanism of inflammasome assembly with ASC between human and mouse NLRP1. NLRP3, NLRP6, and NLRP9 have the same domain structure comprising an N-terminal PYD, a NACHT, and C-terminal LRRs (Figure 4A); however, they have different amino acid lengths and LRR numbers. NLRP3, NLRP6, and NLRP9 are 1036, 892, and 991 amino acids in length and have nine, six, and six LRRs, respectively. NLRC4 has a domain structure similar to that of NLRP3, NLRP6, and NLRP9, but has CARD instead of PYD at the N-terminus (Figure 4A). Non-NLR family PRRs include pyrin and AIM2, which form canonical inflammasomes, as well as mouse caspase-11, and human caspase-4 and -5, which form non-canonical inflammasomes. Pyrin has a different domain structure compared to that of NLR family PRRs and consists of an N-terminal PYD, a B-box-type zinc finger (Bbox), a coiled-coil (CC), and a C-terminal B30.2, which includes two subdomains, PRY and SPRY (Figure 4A). AIM2 is a member of the IFN-inducible p200 protein family [90], and consists of an N-terminal PYD and a C-terminal hematopoietic IFN-inducible nuclear protein 200 (HIN200) (Figure 4A). Caspase-11 was unexpectedly discovered as an intracellular PRR in mice [91,92,93], and its homolog was not initially identified in humans. Later, much effort has demonstrated that human caspase-4 and caspase-5 are the homologs of mouse caspase-11 [94]. Mouse caspase-11, human caspase-4, and caspase-5 have the same domain structure comprising an N-terminal CARD, a p20, and a C-terminal p10 (Figure 4A), but their molecule sizes are different. Mouse caspase-11, human caspase-4, and caspase-5 are 373, 377, and 434 amino acids in length, respectively [10].

The PRRs recognize different types of PAMPs and DAMPs inside the cells in a PRR-specific manner, and are subsequently activated by assembling inflammasomes [7]. Immediately after the sensing of ligands by PRRs, NLR family, pyrin, and AIM2 PRRs assemble canonical inflammasomes via interacting with pro-caspase-1 with or without the help of ASC (Figure 4B) [7,85]. However, mouse caspase-11, human caspase-4, and caspase-5 directly sense their common ligand, intracellular lipopolysaccharide (LPS), which leads to the assembly and activation of non-canonical inflammasomes without the recruitment of pro-caspase-1 and ASC (Figure 4B) [7,79]. The structure and activating ligands of inflammasomes are summarized in Table 1.

### 3.2. Inflammasome-Activated Inflammatory Signaling

As discussed earlier, PRRs recognize the unique types of ligands in a PRR-specific manner (Table 1), and the architecture and mechanism of inflammasome formation are different from one another (Figure 4B). However, inflammasomes share downstream signaling pathways during inflammasome-activated inflammatory responses. Canonical inflammasomes in response to ligands have a molecular architecture that interacts with an inactive form of pro-caspase-1 with or without an adaptor, ASC, leading to the activation of pro-caspase-1 by autoproteolysis removing CARD and the generation of the active form of caspase-1 consisting of p20–p10 dimers [7,79]. The activated caspase-1, in turn, promotes two downstream inflammatory events. Caspase-1 promotes proteolysis of gasdermin D (GSDMD) at Asp276 residue, producing N-terminal (N-GSDMD) and C-terminal fragments (C-GSDMD). The N-GSDMD then moves to the cell membranes and forms the GSDMD pores in the membranes, leading to GSDMD pore-mediated pyroptosis, an inflammatory form of cell death [7,79]. Caspase-1 also facilitates the maturation and activation of the inactive pro-forms of pro-inflammatory cytokines, such as pro-interleukin (pro-IL)-1β and pro-IL-18 by the proteolytic cleavage of N-terminal propeptides and produces the active forms of IL-1β and IL-18, which are secreted from the cells through the GSDMD pores [7,79].

Although the PRRs in canonical inflammasomes recognize a variety of ligands and form inflammasomes with pro-caspase-1 and ASC, the PRRs in non-canonical inflammasomes are activated by very limited types of ligands, and assemble inflammasomes without interacting with pro-caspase-1 and ASC. LPS, an endotoxin of Gram-negative bacterial cell walls, is the first ligand to be identified to activate non-canonical inflammasomes. Mouse caspase-11, human caspase-4, and caspase-5 recognize intracellular LPS internalized by Gram-negative bacteria through direct interaction [7,8,9,10,95,96,97]. Recent studies have identified new ligands, such as lipophosphoglycan (LPG) of *Leishmania* parasites, the oxidized form of endogenous phospholipids, 1-palmitoyl-2-arachidonoyl-sn-glycero-3-phosphorylcholine (oxPAPC), the secreted aspartyl proteinases of *Candida albicans* to activate non-canonical inflammasomes and inflammatory responses [98,99,100]. Despite the discovery of these new ligands, the molecular mechanisms by which these ligands activate non-canonical inflammasomes are poorly understood, and most studies demonstrating the non-canonical inflammasome-activated signaling pathways have been mainly focused on LPS.

To trigger the activation of non-canonical inflammasomes, the LPS of Gram-negative bacteria should enter the cells to interact with caspase-4/5/11 and activate non-canonical inflammasomes. Recent studies have demonstrated that extracellular LPS is internalized by bacterial outer membrane vesicles (OMVs), clathrin, caveolin, lipid rafts, and endocytosis with the help of receptors, such as TLR4, the receptor for advanced glycation end-product (RAGE), and syndecan-1 [101,102,103,104,105]. Internalized LPS by endocytosis is still in the endosomes and should be released from the endosomes to activate non-canonical inflammasomes. Guanylate-binding proteins (GBPs), members of the GTPase family whose expression is increased by IFN, bind to the LPS-containing endosomal membranes and modify the stability of the membranes. This leads to disruption of the endosomal membranes and LPS release from the endosomes to the cytosol to be exposed to caspase-4/5/11 [106,107,108].

LPS internalized and exposed in the cytosol directly interacts with caspase-4/5/11 through its lipid A moiety to the caspase CARD, and LPS-caspase-4/5/11 complexes form caspase-4/5/11 non-canonical inflammasomes by oligomerization via CARD-CARD interaction, resulting in the activation of non-canonical inflammasomes [7,8,9,10,89,90,91]. The activated non-canonical inflammasomes induce the proteolytic cleavage of GSDMD, leading to GSDMD pore formation in cell membranes and GSDMD pore-mediated pyroptosis [7,8,9,10,89,90,91]. It is still unclear whether non-canonical inflammasomes directly induce caspase-1 activation and caspase-1-mediated maturation and secretion of IL-1β and IL-18. Recent studies have reported the functional cooperation between non-canonical and canonical inflammasomes during inflammatory responses and demonstrated that non-canonical inflammasomes activate the NLRP3 canonical inflammasome by facilitating K^+^ efflux through P2X7, THIK1/TWIK2, pannexin-1, and GSDMD pores in membranes, which are key and essential determinants of NLRP3 inflammasome activation [11,109,110]. This then leads to the proteolytic activation of caspase-1 and the caspase-1-mediated maturation and secretion of IL-1β and IL-18 through GSDMD pores [7,8,9,10,89,90,91]. A schematic summary of inflammasome-activated inflammatory signaling pathways is shown in Figure 4C.

## 4. Functional Interplay between Methyltransferases and Inflammasomes

### 4.1. Roles of DNMTs in Inflammasome Functions

DNMTs catalyze DNA methylation, which plays diverse roles in various biological functions and diseases [111]. Interestingly, recent studies have reported the regulatory roles of DNMT-mediated DNA methylation in inflammasome functions, which provides clues that DNMTs might be critical modulators in inflammasome-mediated inflammatory responses and diseases.

Tang et al. investigated the role of DNMT3B in glycolic acid (GA)-mediated anti-inflammatory responses by inhibiting the expression of inflammasome complex genes in human keratinocytes and HaCaT cells. This study reported that while the expression of NLRP3, NLRC4, AIM2, and ASC genes was decreased by GA, GA increased the protein expression and activity of DNMT3B, resulting in the hypermethylation of the promoters of NLRC4 and ASC genes, resulting in the downregulation of the expression of these genes in HaCaT cells [112]. This study suggests that DNMT3B plays an anti-inflammatory role in inflammasome-induced inflammatory responses by inhibiting the expression of inflammasome complex genes, such as NLRC4 and ASC, via hypermethylation of these gene promoters. This study also demonstrates the potential of GA as an anti-inflammatory agent that targets inflammasomes by activating DNMT3B.

Wei et al. reported that NLRP3 inflammasome activation was regulated by DNMT *Sss I*-induced methylation in *Mycobacterium tuberculosis* (Mtb)-infected human monocytes, THP-1 cells. NLRP3 promoter activity was decreased by DNMT *Sss I*-induced methylation, leading to the downregulation of NLRP3 expression, while the inhibition of NLRP3 promoter methylation by DNMT inhibitor upregulated NLRP3 expression in THP-1 cells [113]. In addition, the promoter of the NLRP3 gene was demethylated by Mtb infection, resulting in the upregulation of NLRP3 genes in THP-1 cells [113]. These results indicate that demethylation of the NLRP3 gene promoter by DNMT *Sss I* leads to the inhibition of NLRP3 inflammasome activation by decreasing NLRP3 expression, and Mtb infection promotes inflammasome-induced inflammatory responses by suppressing DNMT *Sss I*-induced methylation of inflammasome gene promoter in human monocytes.

Haldar et al. reported chemotherapeutic-induced DNA damage by global methylation and silencing of DNA damage repair genes in hemorrhagic cystitis, an inflammatory and ulcerative bladder condition. Systemic treatment with chemotherapeutics, cyclophosphamide (CPX), facilitated the accumulation of DNA damage by downregulating the expression of the DNA damage repair gene, Ogg1, and induced subsequent NLRP3 inflammasome-activated pyroptosis of bladder muscle cells in CPX-treated mice [114]. Interestingly, DNMT1 and DNMT3B methylated the promoter of the Ogg1 gene, leading to the Ogg1 silencing and the induction of NLRP3 inflammasome-activated inflammatory responses [114]. Inhibition of Ogg1 silencing by DNA de-methylation suppressed NLRP3 inflammasome activation and NLRP3 inflammasome-induced inflammatory responses, such as pyroptosis and IL-1β secretion in bladder muscle cells [114]. This study is the first to demonstrate the mechanism by which Ogg1 silencing by DNMT-mediated methylation induces NLRP3 inflammasome-activated inflammatory responses in bladder muscle cells and also provides a functional relationship between DNMTs and inflammasomes in systemic chemotherapeutics-induced DNA damage and inflammatory and ulcerative bladder disease, hemorrhagic cystitis.

Huang et al. investigated the DNA methylation levels of NLR family inflammasomes and subsequent inflammatory responses in KD patients with Kawasaki disease (KD). The expression of NLR family PRRs, such as NLRC4 and NLRP12, and pro-inflammatory cytokine, IL-1β, was increased, but the methylation levels of the CpG sites of these genes were much lower in the white blood cells of the KD patients than in the normal control subjects [115], indicating that the hypomethylation of the genes of the inflammasome PRRs, NLRC4, and NLRP12 and pro-inflammatory cytokine IL-1β plays a regulatory role in the upregulation of the expression of these genes, resulting in the induction of inflammatory responses and the pathogenesis of KD.

Zhong et al. reported the regulatory role of DNMT1 in microRNA (miR) expression and inflammasome activation in atherosclerosis in ApoE knockout (KO) mice. DNMT1 hypermethylated the promoter of miR-145, which decreased plaque formation and consequently reduced the expression of miR-145 in vessels, leading to the activation of NLRP3 inflammasome and subsequent inflammatory responses, including the production and secretion of IL-1β in ApoE KO mice [116]. This study provides insights linking DNA methylation and inflammasome activation in atherosclerosis and also provides the potential for developing new therapeutics by targeting DNMT and inflammasomes for the treatment of atherosclerosis as well as other inflammatory diseases.

Sun et al. explored the functional relationship between DNA methylation and inflammasome activation in osteoarthritis (OA) using human osteoarthritic cells and OA patients. The C-terminal-binding proteins (CtBPs) that are highly expressed in OA promote the activation of the NLRP3 inflammasome and downstream inflammatory signaling, such as caspase-1 activation and IL-1β maturation in osteoarthritic cells and OA patients [117]. Interestingly, the expression levels of DNMT1 and DNMT3A were lowered in OA patients, and the knockdown of DNMT1 and DNMT3A resulted in the hypomethylation of CtBP promoters, leading to CtBP overexpression and NLRP3 inflammasome activation in OA patients [117]. These results indicate that DNMTs play an inhibitory role in inflammasome activation and inflammatory responses, whilst also providing insight into the functional relationship between DNMTs and inflammasomes in the pathogenesis of OA.

Zhai et al. reported on the role of *O*^6^-methylguanine-DNA methyltransferase (MGMT), an enzyme that repairs drug-induced DNA damage in acquired drug resistance by modulating inflammasome activation in melanoma cell lines. Melanoma cells treated with temozolomide (TMZ) over two months upregulated the expression of MGMT and became TMZ resistant [118]. Furthermore, TMZ-resistant melanoma cells showed increased expression of NLRP1 and activation of the NLRP1 inflammasome, leading to the maturation and secretion of IL-1β [118]. Although inflammation has been demonstrated to be associated with drug resistance in various cancers in previous studies, this study provides a critical clue for the functional interplay between DNMT and inflammasome in the development of acquired drug resistance in cancers. It also provides invaluable insight into the development of anti-cancer therapeutics against drug-resistant cancers via selective targeting of DNMTs or inflammasomes.

Abplanalp et al. demonstrated the functional cooperation between DNMT mutations and inflammasome activation in the alteration of immune cells in chronic heart failure. Monocytes derived from chronic heart failure patients carrying DNMT3A mutations revealed a significantly increased expression of inflammasome genes, such as NLRP3 and IL-1β, compared with the monocytes isolated from chronic heart failure patients with no DNMT3A mutations [119]. DNMT3A silencing in monocytes also increased the secretion of pro-inflammatory cytokines [119]. Interestingly, monocytes of DNMT3A mutation carriers showed increased expression of T-cell-stimulating molecules and the changes in signatures of T-cell subsets, such as T_H_1, T_H_2, T_H_17, CD4+ memory, CD8 cytotoxic, and regulatory T cells [119]. These results suggest that the monocytes and T-cells with clonal hematopoiesis-driver mutations in DNMT3A play a cooperative role in inducing inflammatory responses in chronic heart failure by promoting the production of a highly inflamed transcriptome and inflammasome activation that may lead to the exacerbation of chronic heart failure.

**Figure 5:**
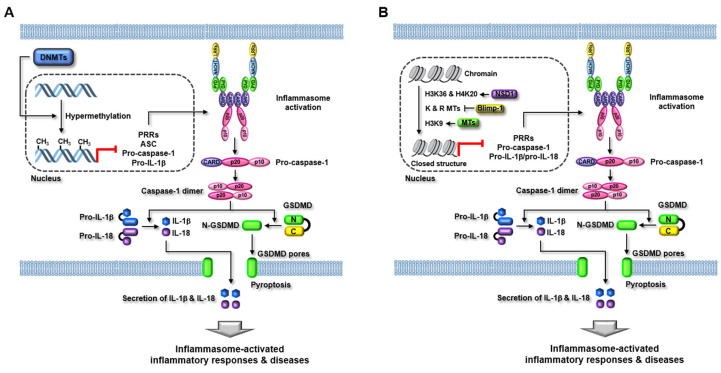
Regulatory role of MTs in inflammasome activation in inflammatory responses and diseases. (**A**) DNMTs induce hypermethylation in the promoter regions or genes of inflammasome components, such as PRRs, ASC, pro-caspase-1, and pro-inflammatory cytokines, and consequently inhibit the transcriptional expression of these genes and inflammasome activation. As a result, the caspase-1-mediated GSDMD pore formation and pro-inflammatory cytokine secretion are subsequently inhibited, resulting in the suppression of inflammasome-activated inflammatory responses and diseases. (**B**) NSD1, Blimp-1, and histone MTs directly or indirectly induce the hypermethylation or hypomethylation of histone proteins at lysine (K) and arginine (R) residues and close the chromatin structure, leading to the inhibition of the transcriptional expression of inflammasome component genes and inflammasome activation. As a result, the caspase-1-mediated GSDMD pore formation and pro-inflammatory cytokine secretion are subsequently suppressed, resulting in the amelioration of inflammasome-activated inflammatory responses and diseases. MTs, methyltransferases; DNMTs, DNA methyltransferases, ASC, apoptosis-associated speck-like protein containing a caspase recruitment domain; GSDMD, gasdermin D.

Yu et al. reported ethanol-induced inflammasome activation and inflammatory responses by modulating the methylation of DNA encoding fat mass and obesity-associated protein (FTO), the m^6^A demethylase in alcohol-induced kidney injury. Ethanol administration induces NLRP3 inflammasome activation, the production of pro-inflammatory cytokines, and renal inflammation in the alcoholic kidneys of mice and human kidney tubular epithelial HK2 cells [120]. Interestingly, ethanol administration highly methylated the DNA encoding FTO and consequently downregulated its expression in the alcoholic kidneys of mice and HK2 cells [120]. Moreover, inhibition of DNMTs, such as DNMT1, DNMT3A, and DNMT3B by their specific inhibitor, 5-azacytidine, recovered FTO expression and alcohol-induced kidney injury in mice and HK2 cells, indicating that alcohol promotes FTO methylation through DNMTs [120]. Moreover, FTO promoted PPAR-α m^6^A methylation and PPARα-induced NLRP3 inflammasome activation in the alcoholic kidneys of mice and HK2 cells [120]. This study suggests that alcohol induces inflammasome activation and renal inflammation by increasing the expression of DNMTs and inducing the subsequent epigenetic modification of FTO and PPAR-α in the kidney. This study also provides a functional association between DNMTs and inflammasomes in alcoholic kidney disease. The regulatory roles of DNMTs in inflammasome function during inflammatory responses and diseases discussed in this study are described in Figure 5 and summarized in Table 2.

### 4.2. Roles of Histone MTs in Inflammasome Functions

Histone proteins have been demonstrated as the major substrates of PMTs [27,28,29]. Histone methylation can be generated at various sites in histone proteins primarily at lysine and arginine residues [121]. Histone methylation can be governed by various positive and negative modulators, resulting in activation or repression of gene transcription [121]. Histone methylation is essential for ensuring the subsequent coordinated transcriptional regulation of gene networks that are critical for normal animal development and almost all biological events—inappropriate histone methylation can cause multiple human diseases [121,122,123]. Recently emerging studies have demonstrated the regulatory roles of histone MTs in inflammasome activation, suggesting that, similar to DNMTs, histone MTs also cooperate with inflammasomes and play regulatory roles in inflammasome-induced inflammatory responses and diseases.

The nuclear receptor-binding SET domain protein 1 (NSD1) is a lysine methyltransferase that preferentially methylates histone 3 (H3) and H4 on lysine 36 residue (K36) and K20, respectively and alters transcription by interacting with the protein NSD1-interacting zinc finger protein 1 (NIZP1) [124,125,126]. Sakhon et al. demonstrated the regulatory role of NSD1 in the activation of caspase-1, a downstream effector of inflammasome activation and caspase-1-mediated inflammatory responses during *Listeria monocytogenes* listeriolysin O (LLO) stimulation of macrophages. LLO induced NLRP3 inflammasome activation and IL-1β secretion and upregulated NSD1 expression in mouse bone marrow-derived macrophages (BMDMs) [127]. Unexpectedly, NSD1 inhibited NLRP3 inflammasome-induced maturation and secretion of IL-1β and IL-18, but neither restricted NLRP3 inflammasome activation at the chromatin level nor influenced NLRP3 gene expression in the LLO-stimulated BMDMs [127]. Interestingly, NSD1 inhibition induced caspase-1 activation and IL-1β secretion in the LLO-stimulated BMDMs [127]. This study suggests a functional association between histone MT, NSD1, and inflammasome signaling during bacterial infection-induced inflammatory responses and further provides the therapeutic potential of infectious diseases by selectively targeting or modulating histone MTs and inflammasome signaling pathways during inflammasome-mediated inflammatory responses.

B lymphocyte-induced maturation protein-1 (Blimp-1) is a DNA-binding zinc finger-containing transcriptional repressor that induces promoter silencing by recruiting histone lysine MTs, histone arginine MTs, histone deacetylase, and co-repressors [128,129,130]. Shi et al. reported the functional crosstalk between the TLR4-Blimp-1 axis and NLRP12 inflammasome in inflammatory responses in dendritic cells and BMDMs, as well as an experimental mouse colitis model. Dextran sodium sulfate (DSS) stimulation induced caspase-1 activation and IL-1β secretion, and inhibition of NLRP12 increased IL-1β secretion in dendritic DC2.4 cells and BMDMs [131]. DSS-induced overexpression of Blimp-1 resulted in the downregulation of NLRP12 expression in DSS-stimulated DC2.4 cells and BMDMs [131]. Moreover, TLR4 was implicated in Blimp-1 upregulation, which subsequently leads to Blimp-1-mediated NLRP12 downregulation and IL-1β secretion in DSS-induced colitis mice [131]. These results indicate that Blimp-1 recruiting and modulating histone MT functions induces inflammatory responses and colitis by inhibiting NLRP12 inflammasome activation in dendritic cells and macrophages. This also suggests evidence of the negative correlation between histone MTs and inflammasomes during inflammatory responses and diseases.

Papale et al. also demonstrated a functional correlation between the NLRP12 inflammasome and Blimp-1 in allergic contact dermatitis in human skin. Contact allergens, such as p-phenylenediamine (PPD) and 2,4-dinitrochlorobenzene (DNCB), increased the expression of Blimp-1 and IL-18, which are critical players in allergic diseases [132], but decrease NLRP12 expression in human keratinocytes, NCTC 2544 cells [133]. This result is in accordance with the previous study by Shi et al. [131], which showed that NLRP12 and Blimp-1 expression is inversely correlated, further demonstrating that Blimp-1 silencing increased NLRP12 expression and reduced contact allergen-induced IL-18 production in NCTC 2544 cells [133]. These results indicate that contact allergens induced allergic contact dermatitis by producing IL-18 through differentially modulating the expression of NLRP12 and Blimp-1 in keratinocytes. It also suggests that histone MTs recruited by Blimp-1 to NLRP12 promoter may play a pivotal role in the inhibition of NLRP12 inflammasome activation and the production of allergic IL-18 in the skin. Furthermore, the study by Shi et al. study [131] provides insight into the development of potential therapeutics for allergic diseases by selectively modulating NLRP12 inflammasome and Blimp-1 activities. Although Blimp-1 suppressed the transcription of inflammasome components by inhibiting the recruitment of histone arginine MTs in these two studies, these studies did not provide the direct evidence that histone arginine MTs are critical players by actively participating in the transcriptional control of inflammasome components, which demands the further studies demonstrating the regulatory roles of histone arginine as well as lysine MTs in transcriptional regulation of inflammasome genes.

IFN-γ-inducible protein 16 (IFI16) is an intracellular sensor of foreign DNA that induces inflammasome-mediated inflammatory responses [134,135,136]. A previous study demonstrated that IFI16 inhibition resulted in the disruption of Kaposi’s sarcoma-associated herpesvirus (KSHV) latency and induced lytic transcript [137], but the underlying molecular mechanism is unknown. Roy et al. investigated the mechanism of IFI16-mediated transcriptional regulation of the KSHV lytic pathway. The study reported the regulatory role of two different histone MTs that methylate H3K9, SUV39H1, and GLP by epigenetic modification of KSHV genomes in IFI16-induced inflammasome responses during host defense immunity. IFI16 interacts with SUV39H1 and GLP to generate the IFI16/SUV39H1/GLP complex, and the complex is recruited to the KSHV genome, leading to the methylation of H3K9 during viral infection and latency [138]. The methylated H3K9 serves as a docking site for HP1α, a heterochromatin-inducing factor, resulting in IFI16-mediated H3K9-trimethylation and silencing of KSHV lytic genes [138]. This study suggests that IFI16 cooperation with histone MTs is one of the critical mechanisms by which IFI16 regulates host innate immunity by sensing foreign DNA and inducing epigenetic modification during viral infection. However, this study did not show direct evidence of how the cooperation between IFI16 and histone MTs regulates the inflammasome signaling pathway in host cells against viral infection, which raises the demand for future studies in this regard. The regulatory roles of histone MTs in inflammasome function during inflammatory responses and diseases discussed in this study are described in Figure 5 and summarized in Table 2.

## 5. Conclusions

Although inflammation is essential for protecting the body from invading pathogens and cellular danger signals, chronic inflammation has been considered as a major risk factor and secret killer for a variety of human diseases. For this reason, much effort has been made to elucidate the mechanisms of inflammatory responses and development of therapeutics against human inflammatory diseases. Between the two steps of inflammatory responses, triggering has been regarded as the critical step in activating and further boosting the inflammatory responses by activating intracellular inflammatory protein complexes, inflammasomes, and subsequent inflammasome-induced effector responses, such as pyroptosis and release of pro-inflammatory cytokines. This has led to extensive studies demonstrating the regulatory roles of inflammasomes in inflammatory responses and diseases. However, despite efforts over the last several decades, it is still under active investigation how inflammasomes are regulated and cooperate with other molecules during inflammatory responses.

The methylation of cellular molecules, such as DNA and proteins, is critical for regulating gene expression and protein activity in various cellular biological functions. In addition, the methylation of non-histone proteins, lipids, and other cellular molecules plays a pivotal role in modulating various cellular functions. Many research groups have therefore investigated the regulatory roles of methyltransferases in multiple physiological and pathological conditions, and have demonstrated the active involvement of DNMTs and PMTs in various biological processes and human diseases by the epigenetic modification of gene promoters and proteins, especially histones. Moreover, studies have attempted to selectively target methyltransferases to develop effective therapeutics for human diseases.

The important role of MT methylation in the regulation of inflammasome functions has gained attention due to the functional cooperation of MTs with inflammasomes in inflammatory responses. This understanding is crucial to the development of novel anti-inflammatory therapeutics by targeting MTs and inflammasomes. Given the evidence discussed in this study, various DNMTs and PMTs, especially histone MTs, play critical roles in regulating inflammasome functions and inflammasome-activated downstream effects in inflammatory responses and diseases. However, despite these successful studies, the regulatory roles of MTs in inflammasome functions have been demonstrated, focusing only on several types of DNMTs and histone MTs, and the functional cooperation between other types of MTs, such as MTs of non-histone proteins, lipids, and other cellular molecules, is poorly understood. In addition, most of the studies have mainly focused on NLRP3 inflammasome and other NLR family inflammasomes—further studies are required to investigate the regulatory role of MTs in the functioning of other types of inflammasomes, especially non-canonical inflammasomes that were recently discovered and demonstrated to play a crucial role in infection-mediated inflammatory responses and diseases.

In conclusion, MTs are critical players in inflammasome functions by the epigenetic modification of DNA and proteins in inflammatory responses and diseases. The selective modulation of MT and inflammasome functions could be a potential strategy to develop novel anti-inflammatory therapeutics to prevent and treat inflammatory and inflammation-mediated diseases.

## Figures and Tables

**Table 1:** Summary of the structure and activating ligands of inflammasomes.

Categories	PRRs	PRR Domains	Inflammasome Components	Activating Ligands
Canonical	NLRP1	PYD, NACHT, LRRs, FIIND, CARD	NLRP1, ASC, pro-caspase-1	*Bacillus anthracis* toxin
	NLRP3	PYD, NACHT, LRRs	NLRP3, ASC, pro-caspase-1	Bacteria, protozoans, viruses, fungi, ATP, cell volume fluctuation, cGAMP, cardiolipin translocation, dsRNA, ER stress, imiquimod, K^+^ efflux, mitochondrial DNA, mitochondrial ROS, particulate matter, lysosomal rupture, RNA–DNA hybrids, nucleic acid hybrids, pore-generating toxins, hyaluronan, extracellular β-amyloids, uric acid, alum, silica
	NLRC4	CARD, NACHT, LRRs	NLRC4, pro-caspase-1	Bacterial needle-like, bacterial flagellin
	NLRP6	PYD, NACHT, LRRs	NLRP6, ASC, pro-caspase-11, pro-caspase-1	Bile acid-derived taurine, lipoteichoic acid
	NLRP9	PYD, NACHT, LRRs	NLRP9, ASC, pro-caspase-1	Short dsRNA
	Pyrin	PYD, Bbox, CC, B30.2	Pyrin, ASC, pro-caspase-1	Bacterial toxin-modified Rho GTPases
	AIM2	PYD, HIN200	AIM2, ASC, pro-caspase-1	dsDNA
Non-canonical	Caspase-4	CARD, p20, p10	Caspase-4, LPS	LPS
	Caspase-5	CARD, p20, p10	Caspase-5, LPS	LPS
	Caspase-11	CARD, p20, p10	Caspase-11, LPS	LPS

**Table 2:** Summary of functional cooperation between MTs and inflammasomes.

Types	Study Results	Exp. Models	Ref.
DNMTs	GA decreased expression of NLRP3, NLRC4, AIM2, and ASC genes in HaCaT cells. GA increased protein expression and activity of DNMT3B in HaCaT cells. GA induced hypermethylation of NLRC4 and ASC gene promoters in HaCaT cells.	Human keratinocytes (HaCaT cells)	[112]
	Mtb infection induced inflammatory responses by increasing NLRP3 gene expression by demethylation of NLRP3 gene promoter in THP-1 cells. NLRP3 promoter activity was decreased by the DNMT *Sss I*-induced methylation, leading to downregulation of NLRP3 expression in THP-1 cells. Inhibition of NLRP3 promoter methylation by DNMT inhibitor upregulated NLRP3 expression in THP-1 cells.	Human monocytes (HP-1 cells)	[113]
	CPX facilitated accumulation of DNA damage by downregulating the Ogg1 gene expression and induced subsequent NLRP3 inflammasome-activated pyroptosis of the bladder muscle cells in the CPX-treated mice. DNMT1 and DNMT3B methylated Ogg1 gene promoter and promoted Ogg1 gene silencing, resulting in induction of NLRP3 inflammasome-activated inflammatory responses in the bladder muscle cells in the CPX-treated mice. Inhibition of Ogg1 silencing by DNA de-methylation suppressed NLRP3 inflammasome activation and NLRP3 inflammasome-induced inflammatory responses (pyroptosis and IL-1β secretion) in bladder muscle cells.	CPX-treated mice Mouse bladder muscle cells	[114]
	Expression of NLRC4, NLRP12, and IL-1β was increased in KD patients. NLRC4, NLRP12, and IL-1β genes were hypomethylated in KD patients.	White blood cells from KD patients	[115]
	miR-145 decreased plaque formation in vessels of ApoE KO mice. DNMT1 induced hypermethylation of miR-145 promoter and decreased miR-145 expression in vessels of ApoE KO mice. DNMT1-mediated downregulation of miR-145 expression induced NLRP3 inflammasome activation and IL-1β secretion in ApoE KO mice.	ApoE KO mice	[116]
	CtBPs are highly expressed in OA. CtBPs promoted activation of NLRP3 inflammasome, caspase-1, and IL-1β in osteoarthritic cells and OA patients. Expression levels of DNMT1 and DNMT3A were lowered in OA patients. Knockdown of DNMT1 and DNMT3A resulted in hypomethylation of CtBP promoters, leading to CtBP overexpression and NLRP3 inflammasome activation in OA patients.	OA patients Human osteoarthritic cells	[117]
	Melanoma cells treated with TMZ over two months upregulated MGMT expression and became TMZ resistant. TMZ-resistant melanoma cells increased NLRP1 expression and induced NLRP1 inflammasome activation, leading to the maturation and secretion of IL-1β.	Human melanoma cells (1205Lu and HS294T cells)	[118]
	Monocytes derived from chronic heart failure patients carrying DNMT3A mutations revealed a significantly increased expression of inflammasome genes, such as NLRP3 and IL-1β compared with monocytes isolated from chronic heart failure patients with no DNMT3A mutations. DNMT3A silencing in monocytes also increased secretion of pro-inflammatory cytokines. Monocytes of DNMT3A mutation carriers showed increased expression of T-cell-stimulating molecules and changes in T-cell signatures.	Monocytes and T-cells from chronic heart failure patients	[119]
	Ethanol administration induced NLRP3 inflammasome activation, pro-inflammatory cytokine production, and renal inflammation in alcoholic kidneys of mice and HK2 cells. Ethanol administration highly methylated FTO DNA and downregulated FTO expression in alcoholic kidneys of mice and HK2 cells. Inhibition of DNMT1, DNMT3A, and DNMT3B recovered FTO expression and alcohol-induced kidney injury in mice and HK2 cells. FTO promoted PPAR-α m^6^A methylation and PPAR-α-induced NLRP3 inflammasome activation in alcoholic kidneys of mice and HK2 cells.	Alcohol-induced kidney injury mice Human kidney tubular epithelial cells (HK2 cells)	[120]
Histone MTs	LLO induced NLRP3 inflammasome activation and IL-1β secretion and upregulated NSD1 expression in mouse BMDMs. NSD1 inhibited NLRP3 inflammasome-induced maturation and secretion of IL-1β and IL-18 in LLO-stimulated BMDMs. NSD1 neither restricted NLRP3 inflammasome activation at the chromatin level nor influenced NLRP3 gene expression in LLO-stimulated BMDMs. NSD1 inhibition induced caspase-1 activation and IL-1β secretion in LLO-stimulated BMDMs.	Mouse BMDMs	[127]
	DSS stimulation induced caspase-1 activation and IL-1β secretion in dendritic DC2.4 cells and BMDMs. Inhibition of NLRP12 increased IL-1β secretion in DSS-stimulated dendritic DC2.4 cells and BMDMs. DSS-induced overexpression of Blimp-1 resulted in downregulation of NLRP12 expression in DSS-stimulated DC2.4 cells and BMDMs. TLR4 expression upregulated Blimp-1 expression and leads to Blimp-1-mediated NLRP12 downregulation and IL-1β secretion in DSS-induced colitis mice.	DSS-treated mice Mouse dendritic cells (DC2.4) and BMDMs	[131]
	Contact allergens increased expression of Blimp-1 and IL-18 and decreased NLRP12 expression in NCTC 2544 cells. Blimp-1 silencing increased NLRP12 expression and reduced contact allergen-induced IL-18 production in NCTC 2544 cells.	Human keratinocytes (NCTC 2544 cells)	[133]
	IFI16 interacted with SUV39H1 and GLP generating the IFI16/SUV39H1/GLP complex. IFI16/SUV39H1/GLP complex was recruited to KSHV genome and induced H3K9 methylation during viral infection and latency. The methylated H3K9 served as a docking site for HP1α, resulting in IFI16-mediated epigenetic modification and silencing of KSHV lytic genes.	KSHV-positive PEL cells (BCBL-1 and BC-3 cells) KSHV-negative BJAB cells	[138]

## Data Availability

Not applicable.

## Abbreviations

PRR	Pattern recognition receptor
PAMP	Pattern-associated molecular pattern
DAMP	Danger-associated molecular pattern
NLR	NOD-like receptor
AIM2	Absent in melanoma 2
MT	Methyltransferase
DNMT	DNA methyltransferase
PMT	Protein methyltransferase
SAM	S-adenosyl-l-methionine
SAH	S-adenosyl-l-homocysteine
PKMT	Protein-lysine methyltransferase
PRMT	Protein-arginine methyltransferase
HMT	Histone methyltransferase
PYD	Pyrin domain
ASC	Apoptosis-associated speck-like protein containing a caspase recruitment domain
CARD	Caspase recruit domain
HIN200	Hematopoietic interferon-inducible nuclear protein 200
LPS	Lipopolysaccharide
GSDMD	Gasdermin D
OMV	Outer membrane vesicle
RAGE	Receptor for advanced glycation end-product
GBP	Guanylate-binding proteins
Blimp-1	B lymphocyte-induced maturation protein-1
